# Opening of St. John's Hospital, Lewisham

**Published:** 1901-06-29

**Authors:** 


### OPENING OF ST. JOHN'S HOSPITAL, LEWISHAM.

ALTHOUGH the new building of the hospital of the sisterhood of St. John the Divine, Morden Hill, Lewisham, has been open for patients since October last year, it did not receive public recognition until Monday, 24th inst.; H.R.H. Princess Christian having been prevented by the death of Queen Victoria from performing the function. She was unfortunately still unable to be present on Monday, but she deputed the Hon. Mrs. TALBOT in her place. The "Office for the Benediction of the Hospital" was said by the Right Rev. the Lord BISHOP of SOUTHWARK, in the men's medical ward, which was unoccupied by patients. The BISHOP then gave a brief address. He recalled the great pleasure with which, not long ago, they had welcomed H.R.H. the Duchess of Albany, who laid the foundation-stone. She had brought a new light into Deptford and the neighbourhood; and when they thought of the increasing population of those parts, with no nearer large hospital than Guy's, it seemed as if they might look forward to a very important future for St. John's Hospital. The founders wanted to steer clear of a difficulty; they did not want to put obstacles in the way of the doctors whose livelihood was earned in this neighbourhood, and they were therefore anxious that the hospital should not be made use of by those patients not absolutely in need of it; it was for the really poor. Such a work needed a great deal of tact and discrimination.

Mrs. Talbot said H.R.H. Princess Christian regretted very much her inability to be present; her interest in hospitals and in everything to do with nursing the sick was very great. She hoped those present would understand why she could not be with them. She would endeavour to respond to their wishes in the future whenever it was possible. It was with her sanction that one of the wards was to be named "Victoria." Mrs. Talbot then declared the hospital open, and proceeded to visit the wards and give each its name.

Returning, the Bishop said it was with H.R.H. Princess Christian's consent that the ward in which the ceremony had taken place was called the "Prince Christian Victor," she had said it would give her the greatest possible pleasure.

